# Case Report: Novel STIM1 Gain-of-Function Mutation in a Patient With TAM/STRMK and Immunological Involvement

**DOI:** 10.3389/fimmu.2022.917601

**Published:** 2022-06-24

**Authors:** Eduardo de la Fuente-Munoz, Ana Van Den Rym, Blanca García-Solis, Juliana Ochoa Grullón, Kissy Guevara-Hoyer, Miguel Fernández-Arquero, Lucía Galán Dávila, Jorge Matías-Guiú, Silvia Sánchez-Ramón, Rebeca Pérez de Diego

**Affiliations:** ^1^ Clinical Immunology Department, San Carlos Clinical Hospital, Madrid, Spain; ^2^ Laboratory of Immunogenetics of Human Diseases, IdiPAZ Institute for Health Research, La Paz University Hospital, Madrid, Spain; ^3^ Innate Immunity Group, IdiPAZ Institute for Health Research, La Paz University Hospital, Madrid, Spain; ^4^ Interdepartmental Group of Immunodeficiencies, Madrid, Spain; ^5^ Neurology Department, San Carlos Clinical Hospital, Madrid, Spain

**Keywords:** STIM1, infection, gain of function, myopathy, calcium signalling

## Abstract

Gain-of-function (GOF) mutations in *STIM1* are responsible for tubular aggregate myopathy and Stormorken syndrome (TAM/STRMK), a clinically overlapping multisystemic disease characterised by muscle weakness, miosis, thrombocytopaenia, hyposplenism, ichthyosis, dyslexia, and short stature. Several mutations have been reported as responsible for the disease. Herein, we describe a patient with TAM/STRMK due to a novel L303P STIM1 mutation, who not only presented clinical manifestations characteristic of TAM/STRMK but also manifested immunological involvement with respiratory infections since childhood, with chronic cough and chronic bronchiectasis. Despite the seemingly normal main immunological parameters, immune cells revealed GOF in calcium signalling compared with healthy donors. The calcium flux dysregulation in the immune cells could be responsible for our patient’s immune involvement. The patient’s mother carried the mutation but did not exhibit TAM/STRMK, manifesting an incomplete penetrance of the mutation. More cases and evidence are necessary to clarify the dual role of STIM1 in immune system dysregulation and myopathy.

## Introduction

Calcium (Ca^2+^) signalling, in which Ca^2+^ acts as a second messenger, controls numerous cellular functions, such as proliferation, apoptosis, exocytosis, differentiation, neurotransmission, hormone secretion, blood coagulation, and muscle contraction. Store*-*operated Ca2*+* entry (SOCE) is a ubiquitous mechanism for Ca2*+* entry into eukaryotic cells mediated by STIM1 and ORAI1 proteins. Human disorders have been related to SOCE, including autosomal recessive STIM1 and ORAI1 loss-of-function (LOF) mutations, resulting in insufficient SOCE and subsequently altering Ca^2+^ release-activated calcium (CRAC) channels (1). The alteration of CRAC channels generates a severe combined immunodeficiency, which includes recurrent and chronic infections, autoimmunity, muscular hypotonia, ectodermal dysplasia, anhidrosis, and mydriasis. Most STIM1 and ORAI1 LOF mutations do not express protein; however, mutations have been described that disrupt the STIM1 function and interfere with the STIM1-ORAI1 interaction (such as R426C and R429C mutations) or generate an obstructed ORAI1 channel, such as the R91W mutation (2). In contrast, gain-of-function (GOF) STIM1 and ORAI1 mutations are autosomal dominant forms that induce overactivation due to excessive Ca^2+^ entry through SOCE. These patients experience tubular aggregate myopathy and Stormorken syndrome (TAM/STRMK), with progressive muscle weakness, myalgia, miosis, ichthyosis, short stature, hyposplenism, thrombocytopaenia (3), and dyslexia (2). All GOF mutations share missense mutations that affect highly conserved amino acids in the EF-hand Ca^2+^-binding motif (H72Q, N80T, G81D, D84G, D84E, S88G, L92V, L96V, Y98C, K104N, F108I, F108L, H109N, H109R, H109Y, I115F), in the sterile alpha motif domain (V138I), in the luminal coiled-coil domains of STIM1 (4) (CC1: R304W and R304Q, and CC2: K365N), between the S/P and K domains (S630F and H632fs*), in the K domain (R749H), and in the ORAI1 transmembrane domains forming the channel pore or concentric rings surrounding the pore (G97C, G98S, V107M, L138F, T184M and P245L) (2, 5–8). Missense mutations in the muscle-specific sarcoplasmic reticulum Ca^2+^ buffer calsequestrin-1 (CASQ1) have been also reported in patients with late-onset muscle weakness and myalgia, forming the mild end of the TAM/STRMK spectrum (9, 10).

## Case Description

We examined a 52-year-old European man from Spain with non-consanguineous parents. Written informed consent was obtained from the patient for the publication of any potentially identifiable data included in this article. The patient had a clear case of TAM/STRMK, with marked myopathy, defective dental enamel, numerous dental caries and root canals, brittle nails, no dystrophy, congenital pes cavus of the right foot, congenital hammer toes (fourth and fifth digits), arthrosis, generalised myalgia, muscle atrophy with myoclonus, and incapacitating fatigue after physical exercise.

The patient also experienced photosensitivity, with erythema and desquamation after sun exposure (doubtful association with drugs); however, there was no report of oral aphthous ulcers, cold sores, arthritis, or Raynaud’s disease. The patient presented skin rashes that worsened in the summer, as well as solar urticaria and dermographism (erythema). He reported that each episode was accompanied by digestive symptoms, with a tendency to diarrhoea and with clinical worsening of his myopathy.

The patient experienced myalgia in the mornings, which decreased with exercise, as well as muscle stiffness, muscle fatigue, myalgia in the peripheral forearms and legs, and muscle contractures that had progressed in the past year. The patient had experienced muscle mass loss even while performing exercise (walking 1 h daily, Pilates), as well as intense post-exercise myalgia. He has always been in good physical shape; however, after 1 hour of exercise, the patient presented fatigue with extreme exhaustion. He woke at night because of muscle pain, which decreased with short walks. The patient experienced myoclonus in the arms with exercise, as well as pain in both wrists, with functional disability and spontaneous resolution.

The patient’s skeletal muscle has preserved architecture but without increased endomysial connective tissue or adipose infiltration. A skeletal muscle biopsy showed discrete variability in muscle fibre size, with the presence of fibres with multiple internalised nuclei (approximately 8%). We observed no necrotic or regenerative fibres and no structural disorders (vacuoles or inclusions). Through ATPase techniques, we determined that the fibre type distribution was normal. With oxidative techniques, we observed a few COX-negative fibres with sorbitol dehydrogenase overexpression (approximately 3%), as well as NADH-TR alterations. We observed no inflammatory infiltrates and no alterations using the histochemical technique for phosphorylase and myoadenylate deaminase. Although the patient’s phosphofructokinase level was not assessable, he presented high creatine kinase levels (**Table 1**) and pseudomyotonia, which was observed on an electromyogram.

**Table 1:** Analytical studies.

		Patient	Healthy controls (range)^d^
**Haemogram^a^** **(without alterations)**	Haemoglobin (g/dL)	14.9	13.1–17.2
	Haematocrit (%)	45.5	39–50
	MCV (fl)	93.5	81–101
	Platelets	227,000	150–450,000
	Leukocytes	4,700	4000–10,000
	Neutrophils	2,000	2000–7000
	Lymphocytes	2,000	1000–3000
**Biochemistry**	Fibrinogen (mg/dL)	379	150–400
	Protein (g/dL)	6.7	6.6–8.3
	Albumin (g/dL)	4.4	3.5–5.3
	Glucose (mg/dL)	100	74–106
	LDH (U/L)	351	208–378
	Creatine kinase (U/L)	736	10–171
	Cholesterol (mg/dL)	275	25–200
**Thyroid profile**	TSH (µU/mL)	1.9	0.3–5.3
	Free T4 (pg/mL)	8.08	5.8–16.4
**Liver profile**	ALT (U/L)	28	3–50
	AST (U/L)	26	3–50
	GGT (U/L)	33	1–55
	AP (U/L)	83	33–120
	Total bilirubin (mg/dL)	0.4	0.3–1.3
**Renal profile**	Creatinine (mg/dL)	1	0.67–1.17
	Estimated glomerular filtration rate (mL/min)	84.9	>60
**Vitamins**	Folic acid (ng/mL)	16.37	3.1–20
	B12 (pg/mL)	254.00	180–914
	Vitamin D (ng/mL)	43.9	30–50
**Complement**	C3 (mg/dL)	122.3	70–140
	C4 (mg/dL)	18.8	15–30
**Immunoglobulins^b^**	IgA (mg/dL)	171	80–400
	IgE (kU/L)	60	0–100
	IgG (mg/dL)	1,084	600–1600
	IgG1 (mg/dL)	612.0	382–930
	IgG2 (mg/dL)	306.1	240–700
	IgM (mg/dL)	172	50–200
**Lymphocyte subpopulations^c^**	CD3 (%) (cells/μL)	70.4 1,198.3	60–83.5 714–2266
	CD4 (%) (cells/μL)	51.71 879.1	32–62 359–1565
	CD8 (%) (cells/μL)	18.45 313.7	11–35 178–853
	CD19 (%) (cells/μL)	9.4 160.4	3–19 61–321
	CD16+ CD56+ (%) (cells/μL)	17.1 291.2	4-18 149–283

^a^Distribution of the patient’s immune cell populations in peripheral blood. Absolute counts ×10^9^ per litre of blood. **^b^** Immunoglobulin levels (IgG, IgG1, IgG2, IgA, IgM and IgE) measured by nephelometry.**^c^** Distribution of lymphocyte subpopulations in the patient’s peripheral blood. **^d^** Internal range.

ALT, alanine aminotransferase; ANAs, antinuclear antibodies; ANCAs, antineutrophil cytoplasmic antibodies; AST, aspartate aminotransferase; AP, alkaline phosphatase; GGT, gamma-glutamyl transferase; Ig, immunoglobulin; LDH, lactate dehydrogenase; MCV, mean corpuscular volume; TSH, thyroid-stimulating hormone.

Beyond the TAM/STRMK disease pattern, the patient presented immunological involvement, with respiratory infections since childhood, chronic cough, and chronic bronchiectasis. At the immunological level, a lymphocyte subpopulation study showed normal T, B, and natural killer cell counts (**Table 1**). The patient had normal activity of complement factors C3, C4, and CH50, as well as normal immunoglobulin levels (**Table 1**).

The patient presented adequate specific antibody response against protein and polysaccharide immunisation. The results of a study of lymphocyte proliferation to mitogens with CD3-CD28 and phytohaemagglutinin were within normal limits. The antibody studies were negative for antinuclear antibodies, antineutrophil cytoplasmic antibodies, celiac antibodies, and *Helicobacter pylori* antibodies.

The acid phosphatase test, and immunohistochemistry for human leukocyte antigen showed no abnormalities. The immunohistochemistry did show preserved membrane proteins (dystrophins 1 and 2, alpha and gamma sarcoglycans, caveolin, and merosin).

The patient showed a good response to the salmonella and tetanus toxoid vaccine. Functional respiratory tests showed reduced baseline spirometry: 11/19 forced vital capacity (FVC) of 4130 mL (77%); forced expiratory volume in 1 second (FEV1) of 3620 mL (91%); and a FEV1/FVC ratio of 88%.

The patient also presented various allergies (pollen, olive trees, reeds, banana, and grasses) and dyslipidaemia, which is under treatment. His allergic symptoms have worsened at the dermatological level, with particular involvement of the chest, back, and legs.

The patient takes eslicarbazepine (800 mg; 0-0-0.5), ezetimibe (10 mg; 0-0-1), chondroitin sulphate (400 mg; 2 doses every 24 h), bilastine (20 mg; 0-1-0), rupatadine (10 mg; 0-0-0-1), and budesonide/formoterol (1-0-1).

## Diagnostic Assessment

The experimental protocol was approved by the ethics committee of Clinico San Carlos University Hospital (Madrid, Spain) and La Paz University Hospital (Madrid, Spain), and written informed consent was obtained from the family for participation in this study.

**Figure 1:**
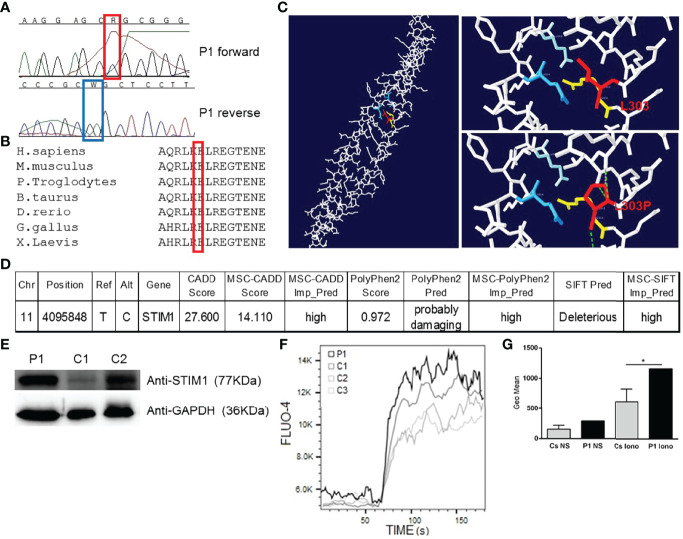
Heterozygous *STIM1* mutation in a patient with myopathy. **(A)** The sequence of the PCR products of genomic DNA from the patient’s leukocytes is shown. g.4095848T>C (c.1477T>C, p.L303P). This mutation has not been previously reported. **(B)** Multiple alignment of the sequences from humans and 6 other species, showing that L303 is a conserved amino acid in 7 analysed species. **(C)** The left panel shows the structure of human CC1-IH STIM1 (4). The top right panel shows the interactions between 2 alpha helices of the CC1-IH region of STIM1. Blue is Q314 and light blue is E318 of one alpha helix, and L303 and R304 are the red and yellow residues, respectively, of the other alpha helix (4). The bottom right panel shows the L303P mutation. The figure was produced using Swiss-PdbViewer. **(D)** Mutation significance cut-off (http://pec630.rockefeller.edu:8080/MSC/) of STIM1 L303P mutation. **(E)** Immunoblot analysis of STIM1 protein from the patient’s (P1) peripheral blood mononuclear cells (PBMCs) and from 2 healthy donors (C1 and C2). We employed GAPDH as a loading control. The panels illustrate the results from a single experiment, representative of 3. **(F)** Calcium flux analysis in the PBMCs of P1 and 3 healthy donors (C1, C2, and C3) in response to ionomycin. The panels illustrate the results from a single experiment, representative of 3. **(G)** Calcium flux geometric mean (Geo Mean) is represented for C1, C2, and C3. ± SD and P1 in non-stimulated PBMCs (NS) or ionomycin-stimulated PBMCs (Iono). *p* < 0.05 (*).

The patient underwent a next-generation sequencing gene panel for the diagnosis of primary immunodeficiencies. We found a variant of the *STIM1* gene on chromosome 11, a heterozygous missense mutation (T/C) affecting the nucleotide position g.4095848 (GRCh37.p13) of exon 7 of the gene encoding STIM1 in the genomic DNA extracted from the leukocytes (g.4095848T>C). This mutation affects leucine at position 303, generating a missense mutation by a proline (c.1477T>C, p.L303P, transcript ID ENST00000300737.4), a previously unreported variant, which we validated by Sanger sequencing of genomic DNA from peripheral leukocytes (**Figure 1A**). His mother, who has cardiomyopathy, has the same *STIM1* mutation (L303P) (data not shown). No more data could be obtained from the mother, the father could not be tested, and the patient has no offspring. No other mutations were found in the *STIM1* coding region. Alignment of the human STIM1 protein sequence with sequences from the 7 animal species in which STIM1 has been sequenced showed L303 to be highly conserved throughout evolution (**Figure 1B**). Protein structure modelling showed how the nonpolar lateral chain of leucine 303 is oriented inside the interaction of the 2 alpha chains of CC1-IH STIM1 (4). The L303P mutation modifies the position of the lateral chain, affecting the proper conformation of the CC1-IH region of STIM1 (**Figure 1C**) (4). A mutation significance cut-off study (http://pec630.rockefeller.edu:8080/MSC/) predicted this variant as likely to be damaging (**Figure 1D**). These data suggest that a heterozygous germline missense *STIM1* mutation (g.4095848T>C; p.L303P) might be responsible for the novel autosomal dominant form of the patient’s GOF STIM1 mutation.

Numerous studies have described the role of *STIM1* mutations in the cells implicated in TAM/STRMK, such as myoblasts and myotubes (5). Given that the patient showed immunological involvement, we wanted to test the protein expression and calcium signalling in immune cells. We then assessed STIM1 protein levels in the patient’s peripheral blood mononuclear cells (PBMCs), detecting normal STIM1 protein levels compared with healthy donors (**Figure 1D**). Given that STIM1 GOF mutations induce excessive Ca^2+^ entry in muscle cells, thereby causing myopathy (10), we wanted to test whether the L303P STIM1 mutation affects calcium homeostasis in PBMCs. We stimulated PBMCs with the calcium ionophore ionomycin, measuring calcium mobilisation by changes in fluorescence of the Fluo4 dye loaded into the cells. Higher calcium flux in response to ionomycin was detected in the patient compared with the 3 healthy donors (**Figures 1E–G**), confirming that the L303P mutation generates a GOF in PBMCs that affects calcium homeostasis. A study of lymphocyte proliferation response to mitogens with CD3-CD28 and phytohaemagglutinin showed normal results (data not shown).

## Discussion

The present case describes a patient with an autosomal dominant GOF *STIM1* mutation responsible for TAM/STRMK. The L303P mutation in the *STIM1* gene showed an incomplete penetrance in the mother, who carried the mutation without TAM/STRMK symptoms. *STIM1* mutations can be divided into those with GOF that manifest TAM/STRMK (2) and those with LOF that have an immune effect responsible for severe combined immunodeficiency, involving recurrent and chronic infections, muscular hypotonia, autoimmunity, ectodermal dysplasia, anhidrosis, and mydriasis (2). However, our patient has a GOF *STIM1* mutation responsible for TAM/STRMK and has had respiratory infections since childhood, including chronic cough and chronic bronchiectasis, despite the main immunological features in terms of immune response and levels of immune cells and other immune parameters being within normal limits. The analysis of immune cells (PBMCs) of the Ca^2+^ signalling revealed a GOF with higher levels of calcium flux compared with healthy donors (**Figures 1E, F**), revealing calcium flux misregulation in the immune cells that could be responsible for the patient’s immune involvement. Worth to mention that the very close residue R304, the only mutated residue described in luminal coiled-coil CC1 domain, in mice harboring GOF R304W mutation presenting TAM/STRMK and abnormal immune cell counts as well as skin abnormalities (11) as shown by our patient.

Despite the study’s limitations resulting from the lack of access to information and studies of the patient’s relatives (incomplete penetrance of the mutation in the mother being the only datum), this is an important clinical case that warrants careful examination to clarify the dual role of STIM1 in immune system dysregulation and myopathy.

## Patient Perspective

It has been widely reported that *STIM1* GOF mutations are responsible for TAM/STRMK and that *STIM1* LOF mutations cause severe combined immunodeficiency. However, it is plausible that a *STIM1* GOF mutation in a patient with TAM/STRMK can also cause immune involvement, given that STIM1 is a protein involved in the immune system and that a misfunction (either by an excess or lack of calcium signalling) would affect the proper functioning of the immune system. Patients with TAM/STRMK should be followed up by clinical immunology departments to find more candidates with whom to study this preliminary finding in more depth. We cannot rule-out the possibility that this immunological involvement would be due to a digenic or polygenic condition by other genes involved in the immune system; however, excess calcium signalling in immune cells highlights a misfunction in the immune system. More cases need to be reported to accumulate sufficient evidence.

## Data Availability Statement

The original contributions presented in the study are included in the article/**Supplementary Material**. Further inquiries can be directed to the corresponding author.

## Ethics Statement 

Study approval: The experimental protocol was approved by the ethics committee of Clinico San Carlos University Hospital (Madrid, Spain) and La Paz University Hospital (Madrid, Spain), and written informed consent was obtained from the family for participation in this study. Written informed consent was obtained from the patient for the publication of any potentially identifiable data included in this article.

## Author Contributions

EF: Physicians in charge of the patient’s care. Clinical report and analytical studies. AR: Calcium signalling and Sanger sequencing. BG-S: Protein expression, Sanger sequencing and protein modelling. JO, KG-H, and MF-A: Clinical study, analytical parameters, manuscript editing. LG: Physician in charge of the patient’s study and care. JM-G: Physician in charge of the patient’s study and care. SS-R: Physician in charge of the patient’s study and care, has read and revised the manuscript. RP: Laboratory head, experiment design, manuscript drafting and editing, Corresponding author. All authors contributed to the article and approved the submitted version.

## Funding

Support was provided by FIS grant Ref. PI17/00543, BG-S is supported by PEJD2019-PRE/BMD-16556 Predoctoral Fellowships CAM and ESID Bridge Fellowship. AR was provided support by FIS grant Ref. PI17/00543.

## Conflict of Interest

The authors declare that the research was conducted in the absence of any commercial or financial relationships that could be construed as a potential conflict of interest.

## Publisher’s Note

All claims expressed in this article are solely those of the authors and do not necessarily represent those of their affiliated organizations, or those of the publisher, the editors and the reviewers. Any product that may be evaluated in this article, or claim that may be made by its manufacturer, is not guaranteed or endorsed by the publisher.
