# Supplementary material for: Case Report: Novel STIM1 Gain-of-Function Mutation in a Patient With TAM/STRMK and Immunological Involvement
Source: Front Immunol. 2022 Jun 24;13:917601. doi: 10.3389/fimmu.2022.917601 (PMC9263075; doi:10.3389/fimmu.2022.917601)
Supplement: Supplementary file 1 [file DataSheet_1.doc]

**This PDF file includes the following sections:**

**Methods**

**Reference**

**METHODS**

**Study approval**

The experimental protocol was approved by the ethics committee of Clinico San Carlos University Hospital (Madrid, Spain) and La Paz University Hospital (Madrid, Spain), and written informed consent was obtained from the family for participation in this study. Written informed consent was obtained from the patient for the publication of any potentially identifiable data included in this article.

**Human molecular genetics and next-generation sequencing gene panel for primary immunodeficiencies**

Genomic DNA was extracted from whole blood with a kit (Qiagen GmbH, Hilden, Germany), according to the manufacturer’s instructions. Targeted next-generation sequencing was performed on genomic DNA from whole blood using a custom capture kit (Sistemas Genomicos). The libraries were sequenced on an Illumina sequencing platform (mean coverage >80 to 100X).

The results of the next-generation sequencing gene panel were validated by polymerase chain reaction (PCR)/sequencing analysis on genomic DNA from whole blood. The PCR was performed with PCR Master Mix (Promega, Fitchburg, WI, USA) and the GeneAmp 9700 PCR System (Applied Biosystems, Foster City, CA, USA). The following primer sequences were employed for exon 7 of STIM1:

Forward primers (FP): 7F, CATGTTGGCTGGCACCCC; Reverse primers (RP): 7R, CCCTTTACCCACCCGTCAA.

The PCR products were purified with ExoSAP*-*IT PCR Product Cleanup Reagent (Applied Biosystems) and sequenced with the BigDye Terminator Cycle Sequencing Kit (Applied Biosystems). Sequencing products were purified by precipitating in 70% ethanol, and the sequences were analysed with an ABI Prism 3700 Genetic Analyser (Applied Biosystems).

Sanger sequencing are deposited in the DNA Data Bank of Japan
Bioinformation (DDBJ) repository (Mishima, Shizuoka 411-8540, Japan): Accession number: LC706737, EntryID: 625fa8883a01a50064596bd0.STIM1L303P.

**Multiple amino acid sequence alignment**

The *homo sapiens* STIM1 (NP_001264890.1) amino acid sequence was aligned with other STIM1 sequences using the Basic Local Alignment Search Tool (BLAST), employing the protein database of the National Center for Biotechnology Information (NCBI, http://blast.ncbi.nlm.nih.gov/Blast.cgi). Multiple sequence alignment was performed with CLUSTAL W2 (a general-purpose DNA or protein multiple sequence alignment program), based on the BCL10 amino acid sequences of *Mus musculus*, *Pan troglodytes,* *Bos Taurus,* Danio rerio, *Gallus gallus*, and Xenopus laevis*.*

**Protein structure modelling**

The CC1-IH region of human STIM1 (4O9B) (1) is represented by Swiss-PdbViewer and produced the mutation L303P.

**Immunoblots**

Human PBMCs were isolated by Ficoll-Hypaque density gradient centrifugation (Amersham-Pharmacia-Biotech, Buckinghamshire, UK) from whole-blood samples obtained from the patient and healthy volunteers, and total cell extracts were prepared. Equal amounts of protein from each sample were separated by SDS-PAGE and blotted onto iBlot Gel Transfer Stacks (Invitrogen). These nitrocellulose membranes were then probed with anti-STIM1 polyclonal antibody (PAC875Hu01, Cloud-Clone Corp., Katy, TX, USA), followed by a secondary anti-rabbit IgG-HRP linked antibody (Cell Signaling, Beverly, MA, USA). Membranes were stripped and re-probed with an antibody against GADPH (Abcam, Cambridge, MA, USA) for protein loading. Antibody binding was detected by enhanced chemiluminescence (Amersham-Pharmacia-Biotech).

**Calcium flux**

Fluo-4 (Molecular Probes, Invitrogen, Carlsbad, CA, USA) was loaded in PBMCs from the patient and healthy donors and then stimulated with 2.5 ng/μL of ionomycin (Sigma, St Louis, MO, USA). Fluorescence measurements were performed using a BD FACSCelesta flow cytometer (BD Biosciences, San Jose, CA, USA), and the data were analysed by FlowJo (Kinetics) (BD). The histograms show the populations gated by forward scatter versus side scatter and singlets. Data are representative of 3 different experiments.

### Statistical analysis

The statistical significance was determined using a t-test (parametric test), comparing the differences between the healthy donors and the patient with the same stimuli. Differences between the samples were statistically significant at p<0.05. The statistical analysis was performed using GraphPad Prism version 8.0.

**REFERENCES**

1. Cui B, Yang X, Li S, Lin Z, Wang Z, Dong C, Shen Y. The Inhibitory Helix Controls the Intramolecular Conformational Switching of the C-Terminus of STIM1*. PLoS O*ne (2013**)** 8:e74735. doi:10.1371/journal.pone.0074735
